# Transitional cell carcinoma of the ovary: A rare case and review of literature

**DOI:** 10.1186/1477-7819-8-98

**Published:** 2010-11-14

**Authors:** EM Tazi, I Lalya, MF Tazi, Y Ahellal, H M'rabti, H Errihani

**Affiliations:** 1 Departement of Medical Oncology, National Institute of Oncology, Rabat, Morocco; 2 Departement of Urology, CHU Hassan II, Fez, Morocco

## Abstract

**Introduction:**

Transitional cell carcinoma (TCC) of the ovary is a rare, recently recognized, subtype of ovarian surface epithelial cancer.

**Case presentation:**

A 69-year-old postmenopausal woman presented with a 2-year history of progressive enlargement of an abdominal mass. Abdominal computed tomography showed a pelvic mass. CA-125 was normal. A staging operation with total abdominal hysterectomy, bilateral salpingo-oophorectomy, infracolic omentectomy and pelvic lymph node dissection was performed. After surgery, the pathologic report of the right ovarian tumour was TCC, grade 3, stage IC. The patient underwent 3 cycles of chemotherapy: carboplatin and paclitaxel. She is regularly followed up and has been disease free for 10 months

**Conclusion:**

Transitional cell carcinoma (TCC) of the ovary is a rare subtype of epithelial ovarian cancer. Surgical resection is the primary therapeutic approach, and patient outcomes after chemotherapy are better than for other types of ovarian cancers.

## Introduction

Transitional cell carcinoma (TCC) of the ovary is a rare, recently recognized, subtype of ovarian surface epithelial cancer. In a study by Silva et al, focal or diffuse TCC pattern was seen in 88 of 934 ovarian cancers [1]. Here, we present a case of TCC of the ovary, managed by total abdominal hysterectomy and bilateral salpingo-oophorectomy with infracolic omentectomy and pelvic lymph node dissection followed by chemotherapy.

## Case presentation

**Figure 1:**
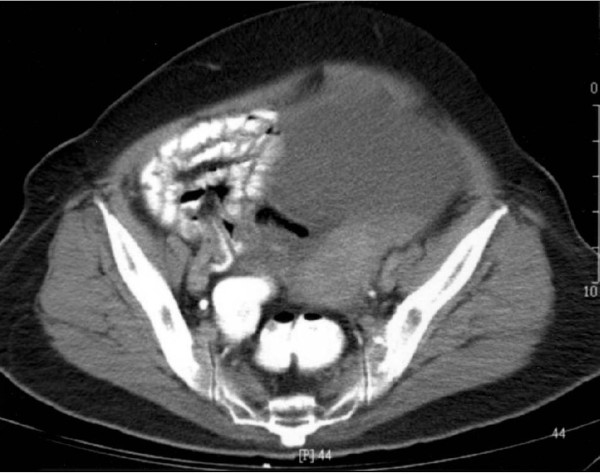
**Abdominal computed tomography shows homogeneous cyst on the right side of the pelvis, which was larger than 35 mm in maximal diameter with a solid component**.

**Figure 2:**
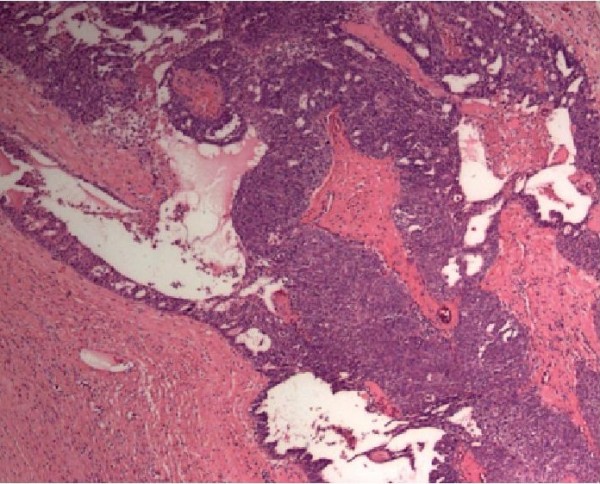
**Ovarian transitional cell carcinoma**. (hematoxylin & eosin, 40×).

**Figure 3:**
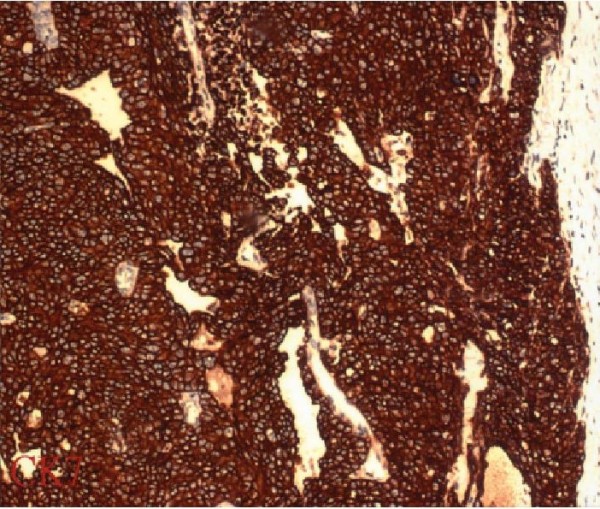
**Immunohistochemical staining of ovarian transitional cell carcinoma**. Tumor cells are positive for cytokeratin 7.

A 69-year-old postmenopausal woman presented with a 2-year history of progressive enlargement of an abdominal mass. She had experienced weight loss of about 4 kg during the 6 months prior to admission. Physical examination showed a pelvic mass. Abdominal ultrasound showed a pelvic mass measuring 31 × 35 mm with homogeneous echogenicity. Abdominal computed tomography (CT) showed a homogeneous cyst on the right side of the pelvis, which was larger than 35 mm in maximal diameter with a solid component. There was no evidence of lymphadenopathy. The liver and kidneys were unremarkable (Figure 1). Routine biologic test results were all within normal ranges. Initial investigation of tumor markers before surgery showed normal serum CA-125 (5.3 U/mL; normal, 0-35 U/mL). She underwent surgery under the impression of malignant ovarian tumor. A small amount of ascites (about 100 mL) in the pelvic cavity was found intraoperatively. A cystic mass, measuring 3, 5 × 1.5 cm, arising from the right ovary; was resected. There was no enlargement of the paraaortic lymph node on palpation. Therefore, surgical staging procedures including total abdominal hysterectomy, bilateral salpingo-oophorectomy, infracolic omentectomy and pelvic lymph node dissection were performed. The ascites was also sent for cytologic examination. Microscopic examination showed malignant transitional epithelial lining of the right ovarian cyst. There was no metastatic lesion and the cytology of the ascites was positive. The final diagnosis was TCC, grade 3, stage IC (Figure 2). Immunohistochemical studies showed that the tumor was positive for cytokeratin 7 and CA 125 (Figure 3) and negative for CK20. The patient received postoperative chemotherapy with carboplatin (area under the curve, 5) and paclitaxel (175 mg/m2) every 3 weeks for three cycles because stage Ic. The patient is being regularly followed up and has been diseasefree for 10 months.

## Conclusions

### Epidemiology and Description

TCC of the ovary is a recently recognized subtype of ovarian surface epithelial cancer. It has been described as a primary ovarian carcinoma in which definite urothelial features are present but no benign, metaplastic and/or proliferating Brenner tumor can be identified. TCC of the ovary was first defined by Austin and Norris [2]. They reported a group of patients who had ovarian tumors presenting with histologic features similar to those seen in a malignant Brenner tumor, but the tumors lacked the associated benign Brenner tumor component. Pure TCC was thus distinguished from malignant Brenner tumor. In addition to not having a benign Brenner tumor component, TCC lacks the prominent stromal calcification [2]. The true incidence of TCC of the ovary remains unknown. Because TCC of the ovary has close morphologic similarities to TCC of the bladder and it behaves more aggressively than malignant Brenner tumor, Austin and Norris concluded that ovarian TCC arises directly from the pluripotential surface epithelium of the ovary and from cells with urothelial potential, rather than from a benign or proliferative Brenner tumor precursor. The metastatic pathways of the tumor are mimicking the transitional cell carcinoma of the bladder wich implicate a loss of the integrity of E-cadherin [2].

### Diagnosis

As described in detail by Eichhorn and Young, ovarian TCC typically showed undulating, diffuse, insular and trabecular growth patterns [3]. The tumor cell nuclei were oblong or round, often exhibiting nucleoli or longitudinal grooves. The cytoplasm was often pale and granular, rarely clear or eosinophilic. The common presenting symptoms of TCC of the ovary are abdominal pain, abdominal swelling or distension, and weight loss. Occasionally, the patient may present with uterine bleeding, back pain, bowel or urinary symptoms. The clinical presentation is indistinguishable from other types of ovarian carcinoma [2,3]. CA-125 is clinically useful as a serum marker of tumor progression and recurrence.

### Histopathology and immunochemistry

The immunophenotype of TCC of the ovary is similar to that of other surface carcinomas of the ovary, but differs from that of TCC of the bladder[1]. In addition, ovarian TCCs are negative for CK20, thrombomodulin (TM) and uroplakin III, which are the antigens that are usually (CK20) or sometimes (TM and uroplakin III) detected in bladder TCCs. Unlike bladder TCCs, ovarian TCCs are often positive for vimentin, CA-125 and Wilms tumor protein (WT1)[3]. Croft et al concluded that almost all of the ovarian TCCs marked strongly for estrogen receptors (ERs), a characteristic that may help to differentiate these lesions from papillary urothelial carcinoma metastatic to the ovary[4]. Shen et al described that overexpression of p53 in TCC of the ovary was associated with a poor prognosis [5]. However, Gershenson et al. concluded that immunostaining for p53, epidermal growth factor receptor, HER-2/neu, DNA ploidy, and S-phase fraction did not distinguish TCC from other common epithelial ovarian cancers[6,7]. TCC of the ovary is reported to be sensitive to cisplatin-based chemotherapy and has a better prognosis than other types of common epithelial tumors of the ovary. Sweeten et al. suggested that TCC may be more chemosensitive than other common epithelial tumors in the refractory setting [8].

### Prognosis

The relative influences of tumor biology and treatment strategies remain undetermined. Gershenson et al. concluded that advanced-stage ovarian TCC was significantly more chemosensitive and associated with better prognosis than poorly differentiated serous carcinoma[9]. Kommoss et al also documented that patients with TCC had better prognoses compared to patients with all other types of ovarian carcinomas after standardized chemotherapy[10].

### Treatment

Optimal surgical resectability followed by cisplatin-based chemotherapy might contribute to the survival benefit [10]. In their study, Silva et al reported that the estimated 5-year survival rate after surgery for 88 patients was 37%, whereas for patients who received chemotherapy, it was 41% [1]. Factors associated with survival for patients who received chemotherapy were the clinical stage, the percentage of TCC component in the primary tumor, and the results of the second-look operation. The predominance of TCC was a favorable prognostic factor and patients with higher clinical stages had poorer prognoses.

## Consent

Written informed consent was obtained from the patient for publication of this case report and accompanying images. A copy of the written consent is available for review by the Editor-in-Chief of this journal.

## Competing interests

The authors declare that they have no competing interests.

## Authors' contributions

ET, IL and HM analyzed and interpreted the patient data regarding its oncological features. MFT and YA have been involved in drafting the manuscript and HE has given final approval of the version to be published. All authors read and approved the final manuscript.
